# Dihydroisotanshinone I combined with radiation inhibits the migration ability of prostate cancer cells through DNA damage and CCL2 pathway

**DOI:** 10.1186/s40360-018-0195-4

**Published:** 2018-01-31

**Authors:** I-Yun Lee, Yin-Yin Lin, Yao-Hsu Yang, Yu-Shin Lin, Chun-Liang Lin, Wei-Yu Lin, Yu-Ching Cheng, Li-Hsin Shu, Ching-Yuan Wu

**Affiliations:** 1 Department of Chinese Medicine, Chiayi Chang Gung Memorial Hospital, No.6, W. Sec., Jiapu Rd., Puzi City, Chiayi County, 613 Taiwan, Republic of China; 2 School of Chinese medicine, College of Medicine, Chang Gung University, Tao-Yuan, Taiwan; 3 Department of Pharmacy, Chiayi Chang Gung Memorial Hospital, Chiayi, Taiwan; 4 Departments of Nephrology, Chiayi Chang Gung Memorial Hospital, Chiayi, Taiwan; 5 Kidney and Diabetic Complications Research Team (KDCRT), Chiayi Chang Gung Memorial Hospital, Chiayi, Taiwan; 6 Department of Urology, Chang Gung Memorial Hospital at Chiayi, Puzi City, Taiwan; 7 Chang Gung University of Science and Technology, Chia-Yi, Taiwan

**Keywords:** Dihydroisotanshinone I, Radiosensitive, Prostate cancer, DNA damage, CCL2

## Abstract

**Background:**

Radiotherapy plays an important role in the treatment of prostate cancer. Despite that sophisticated techniques of radiotherapy and radiation combined with chemotherapy were applied to the patients, some tumors may recur. Therefore, the study investigated the effect of dihydroisotanshinone I (DT) and the combination treatment of 5 μM DT and 5Gy irradiation (IR) against the migration ability of prostate cancer cells.

**Methods:**

DT and the combination treatment were studied for its biological activity against migration ability of prostate cancer cells with transwell migration assay. Subsequently, we tried to explore the underlying mechanism with ELISA, flow cytometry and Western’s blotting assay.

**Results:**

The results showed that DT and the combination treatment substantially inhibited the migration ability of prostate cancer cells. DT and the combined treatment can decrease the ability of macrophages to recruit prostate cancer cells. Mechanistically, DT and the combination treatment reduced the secretion of chemokine (C-C Motif) Ligand 2 (CCL2) from prostate cancer cells. We also found that DT treatment induced the cell cycle of prostate cancer cells entering S phase and increased the protein expression of DNA damage response proteins (*r*H2AX and phosphorylated ataxia telangiectasia-mutated [ATM]) in DU145 cells and PC-3 cells.

**Conclusions:**

DT displays radiosensitization and antimigration effects in prostate cancer cells by inducing DNA damage and inhibiting CCL2 secretion. We suggest that DT can be used as a novel antimetastatic cancer drug or radiosensitizer in the armamentarium of prostate cancer management.

## Background

Radiotherapy is an effective form of local cancer treatment because it induces the DNA damage response (DDR) [1]. However, a fraction of tumors recur after such treatment, usually in more aggressive and metastatic forms [2]. Sensors inside cells can recognize DNA damage and start the DDR process, which induces cell cycle arrest to allow the damaged DNA to be repaired. Among the different types of DNA damage events, DNA double-strand breaks (DDBs) are the most lethal. During DDBs, ATM (previously known as ataxia–telangiectasia mutated) is phosphorylated and activated, serving as a pivotal regulator for the execution of DDR in the maintenance of genomic stability. Another protein, H2AX, acts as an important platform for recruiting DDR proteins. Activated ATM then phosphorylates histone H2AX at S139 (known as *r*H2AX), which recruits a mediator of DNA damage, checking protein 1 (MDC1), to the sites of DNA breaks, which in turn recruits downstream repair proteins to DNA damage foci for repair [3–5]. During DDBs, the S phase can be delayed. Notably, these DDR proteins can be crucial in cancer treatment with chemotherapy agents and radiotherapy. Despite the sophisticated radiation techniques that have been developed, as well as the combination of radiation with chemotherapy, some tumors do recur. Thus, a method that improves the local control of primary or metastasized tumors with a combination of radiotherapy and radiosensitizer may be beneficial for patients with cancer.

**Fig. 1:**
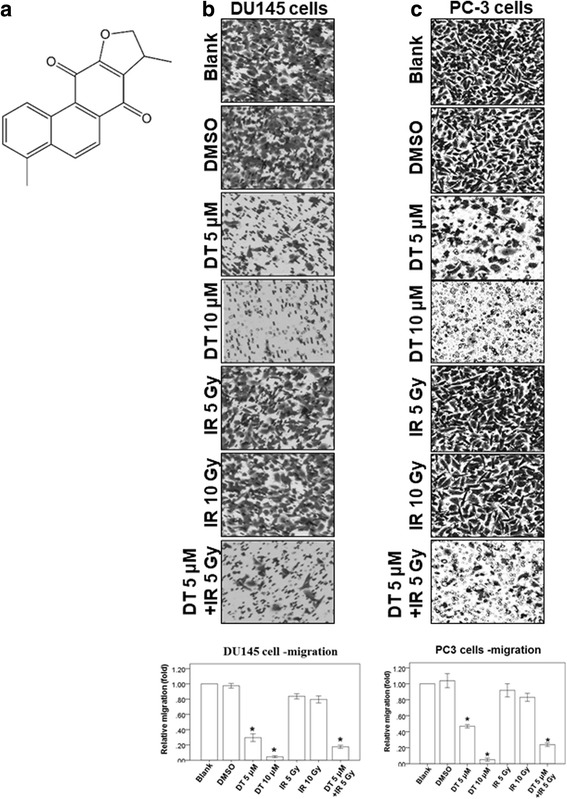
DT blocks different human prostate cancer cells migration on in vitro Transwell migration assay. **a** The structure of dihydroisotanshinone I (DT). **b**, **c**, The migration ability of DU145 cells (**b**) and PC-3 cells (**c**) were measured with the transwell migration assay. After treated with indicated drugs for 24 h, the photographs (× 100) were taken and the migratory cells were measured using AlphaEase®FC StandAlone Software. Numbers of the migratory DU145 cells and PC-3 cells in each group were normalized to the control. The results were from three independent experiments. (Error bar = mean ± S.E.M. Asterisks (*) mark samples significantly different from blank group with *p* < 0.05)

Tumor-associated macrophages are derived from peripheral blood monocytes that are recruited into the tumor and potentiate the seeding and establishment of metastatic cells [6]. C-C motif chemokine ligand 2 (CCL2), also known as monocyte chemoattractant protein-1, was first identified by its ability to attract monocytes in vitro [7, 8]. CCL2 recruits prostate cancer epithelial cells to the bone microenvironment and regulates their rate of proliferation [9, 10]. Dihydroisotanshinone I (DT) (Fig. 1a), a substance extracted from the dried root of Salvia miltiorrhiza Bunge, contains abietane-type diterpene quinone. In a previous study [11], tanshinone IIA inhibited the metastasis of hepatocellular carcinoma and was identified as a potential means of increasing survival rates. In our previous study, we noted that DT substantially inhibited the migratory ability of prostate cancer cells in both a macrophage-conditioned medium and a macrophage/prostate cancer coculture medium [12]. However, the effect of DT combined with radiotherapy on prostate cancer cells and the underlying mechanism remain unclear. In this study, we investigated the effect of DT in combination with ionizing radiation (IR) on the migration of prostate cancer cells in a macrophage medium. We also observed the exact mechanism for combining DT with radiation therapy.

## Methods

### Cell culture and treatment

The human prostate cancer cell lines (DU145 cells and PC-3 cells), human acute monocytic leukaemia cell line (THP-1 cells), IMR-9 cells (human normal lung fibroblast) were obtained from the American Type Culture Collection. The DU145 cells and PC-3 cells were cultured in Dulbecco’s Modified Eagle’s medium (DMEM) (Invitrogen Corp., Carlsbad, CA), supplemented with 10% FBS at 37 °C and 5% CO_2_. The THP1 cells were cultured in RPMI-1640 medium (RPMI) (Invitrogen Corp., Carlsbad, CA), supplemented with 10% fetal bovine serum (FBS) at 37 °C and 5% CO_2_. IMR-90 cells were cultured in Minimum essential medium Eagle (Invitrogen Corp., Carlsbad, CA), supplemented with 10% fetal bovine serum (FBS) at 37 °C and 5% CO2. Recombinant Human CCL2 was obtained from Peprotech. Dihydroisotanshinone I (DT) was obtained from ChemFaces Natural Products Co., Ltd., China (Catalog number: CFN-90162, the purity of dihydroisotanshinone is 98% and its solubility in DMSO is > 5 mg/mL). Human prostate cancer cells and macrophages were cultured to 60–70% confluence prior to treatment. Medium was then replaced with fresh medium containing dihydroisotanshinone in DMSO (dimethyl sulfoxide) at the indicated concentrations. Cells treated with DMSO alone were used as untreated controls.

### Cell migration assay

Cell migration assays were performed as described previously [13]. For the migration of human prostate cancer cells in monoculture, the indicated cell lines (DU145 cells, PC-3 cells, or IMR-90 cells) (1 × 10^5^ cells/well) were plated in the upper chambers of Transwell plates with 8-μm pore polycarbonate membrane inserts in a medium without fetal bovine serum (FBS). A medium with FBS was plated in the lower chambers. After treatment or no treatment with dimethyl sulfoxide (DMSO) and with the indicated treatment for 16–24 h, the cells that had migrated to the bottom were fixed and stained using 1% toluidine blue. The numbers of cells were averaged after six randomly selected fields were counted. For the prostate cancer recruitment assay, THP-1 cells (1 × 10^5^ cells/well) were cultured for 24 h. The THP-1 cell medium was then collected and plated in the lower chambers. After treatment or no treatment with DMSO and with the indicated treatment for 24 h, the human prostate cancer cells (DU145 cells or PC-3 cells) (1 × 10^5^ cells/well) were plated in the upper chambers in the medium without FBS. After incubation for 16–24 h, the cells that had migrated into the bottom were fixed and stained using 1% toluidine blue, and the numbers were averaged after six randomly selected fields were counted. Each sample was assayed in triplicate, and each experiment was repeated at least twice.

### Enzyme-linked immunosorbent assay

Enzyme-linked immunosorbent assays (ELISAs) were performed as described previously [14]. The medium was collected from a monoculture of prostate cancer cells with or without DMSO treatment, the indicated concentration of DT, or the indicated dose of IR for 24 h. Human CCL2 or human interleukin 8 (IL-8) in medium were detected with human CCL2 ELISA kits (eBioscience, catalog number: 88–7399) or human IL-8 ELISA kits (eBioscience, catalog number: 88–8086) according to the manufacturer’s instructions.

### Western blot analysis

Western blot analyses were performed as described previously [15]. Cellular extracts of the human prostate cancer cell line (DU145 cells and PC-3 cells) that had been treated with DMSO or 10 μM of DT for 0 or 6 h were prepared according to the manufacturer’s instructions. Equal amounts of protein were fractionated via 6% or 12% SDS-PAGE and transferred to polyvinylidene difluoride (PVDF) membranes. The membranes were then blocked with 5% nonfat dried milk for 30 min and incubated in primary antibody for 3 h at room temperature. The primary antibodies used were anti-H2AX antibody (Cell Signaling, ratio: 1:1000), antiphosphorylated (S139)-H2AX antibody (Cell Signaling, ratio: 1:1000), anti-ATM antibody (Cell Signaling, ratio: 1:1000), antiphosphorylated (S1981)-ATM antibody (Rockland, ratio:1:2000), and anti-α-tubulin antibody (Santa Cruz, IB: 1:10,000). The primary and secondary antibodies were diluted with 1% nonfat dried milk in 0.1% tris-buffered saline with Tween 20 (TBST). Blots were washed with 0.1% TBST and incubated in horseradish peroxidase-conjugated secondary antimouse or antirabbit antibodies (Santa Cruz, ratio: 1:5000) for 1 h at room temperature. After washing with 1X TBST again, protein signals were detected by chemiluminescence with the SuperSignal Substrate (Pierce, Number: 34,087).

### Cell cycle assay

DU145 cells or PC-3 cells (1 × 10^6^ cells) were seeded in a 100-mm plate and cultured overnight before treatment. The cells were then treated with 10 μM of DT for 0–6 h. After incubation, the cells were analyzed using flow cytometry with propidium iodide (PI) staining to determine the distribution of the G0/G1, G2/M, and S phases of the cell cycle. Briefly, the medium was removed and harvested after washing with phosphate-buffered saline (PBS). The supernatant was removed by centrifugation. Cell pellets were washed with PBS and fixed in 75% ethanol overnight at − 20 °C. The next day, the treated cells were washed with cold PBS and stained with PI solution (50 μg/mL PI [Sigma-Aldrich] and 0.5 μg/mL ribonuclease [Sigma-Aldrich] in PBS) in a 37 °C water bath for 15 min. The cell cycle stages were measured by flow cytometry.

### Statistical analyses

All values were the means ± standard error of mean (SEM) of the replicate samples (*n* = 3 to 6, depending on the experiment) and experiments were repeated by a minimum of three times. Differences between two groups were assessed using the unpaired two-tailed Student’s *t*-test or by ANOVA if more than two groups were analyzed. The Tukey test was used as a post-hoc test in ANOVA for testing the significance of pairwise group comparisons. *P*-values < 0.05 were considered statistically significant in all comparisons. SPSS version 13.0 for windows (LEAD technologies, Inc.) was used for all calculations.

## Results

### DT and the combination treatment can block cell motility in different human prostate cancer cells

To study the effect of DT on the migration ability of prostate cancer cells, we used two prostate cancer cell lines (PC-3 and DU145) to study the function of DT, IR, and the combined therapy with 5 μM DT and 5 Gy IR in the migration assay (Fig. 1b, c). The results showed that 5–10 μM DT considerably inhibited the migration ability of prostate cancer cells in a dose-dependent manner. We also used 5–10 Gy IR to compare the effects of DT on migration. Our results showed that neither 5 nor 10 Gy IR were able to inhibit the migration ability of prostate cancer cells, whereas 10 μM DT was able to do so. Combination treatment with 5 μM DT and 5 Gy IR substantially inhibited the migration ability of prostate cancer cell as 10 μM DT did. These data suggest that DT and the combined therapy can inhibit the migration of prostate cancer cells in an effective manner.

### Effects of DT and the combined therapy on prostate cancer cell migration in the THP-1 cell medium

**Fig. 2:**
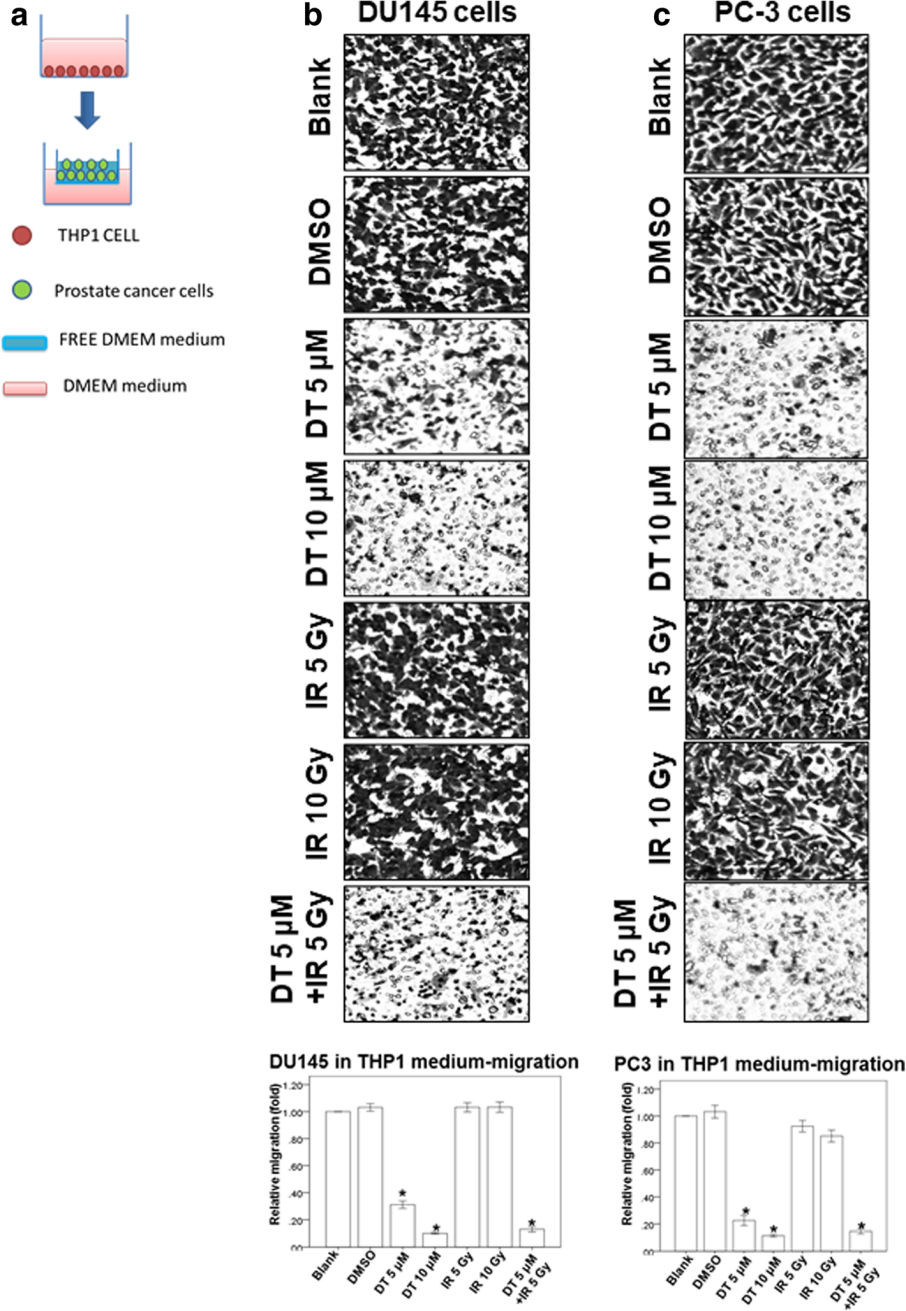
The effect of DT on prostate cancer cell migration in the THP1 cell medium. The migration ability of human prostate cancers in the THP1 cells medium were measured with the transwell migration assay. **a** THP1 cells were cultured for 24 h. Then the condition medium were collected and placed in the lower chamber. **b**, **c** The DU145 cells (**b**) and PC-3 cells (**c**) were then placed on the upper chamber for the migration assay. After incubation for 24 h, the photographs (× 100) were taken and the migratory cells were measured using AlphaEase®FC StandAlone Software. Numbers of the migratory DU145 cells and PC-3 cells in each group were normalized to the control. The results were from three independent experiments. (Error bars = mean ± S.E.M. Asterisks (*) mark samples significantly different from blank group with *p* < 0.05)

Previous studies have shown that macrophages promote tumor invasion and metastasis and that the motility of human prostate cancer cells is inhibited by DT. Therefore, we investigated the effect of DT and the combined therapy on the ability of macrophages to promote tumor migration. Because THP-1 cells are extensively used to study monocyte/macrophage functions, mechanisms, signaling pathways, and drug activities [16, 17], we used THP-1 cells as our model (Fig. 2a). In the control group, DU145 cells and PC-3 cells were recruited by the conditioned medium. This demonstrated that THP-1 cell medium can promote the migration ability of prostate cancer cells. However, 5–10 μM DT markedly inhibited the migration of prostate cancer cells in a dose-dependent manner in the THP-1 cell medium (Fig. 2b, c). Moreover, 5 and 10 Gy IR alone did not inhibit the migration ability of DU145 cells and PC-3 cells. However, combined treatment (5 μM DT plus 5 Gy IR) was able to inhibit the migration ability of prostate cancer cells. These data suggested that DT or combined treatment may diminish the ability of macrophages to recruit prostate cancer cells.

### DT and combined therapy inhibit the secretion of CCL2 by prostate cancer cells

**Fig. 3:**
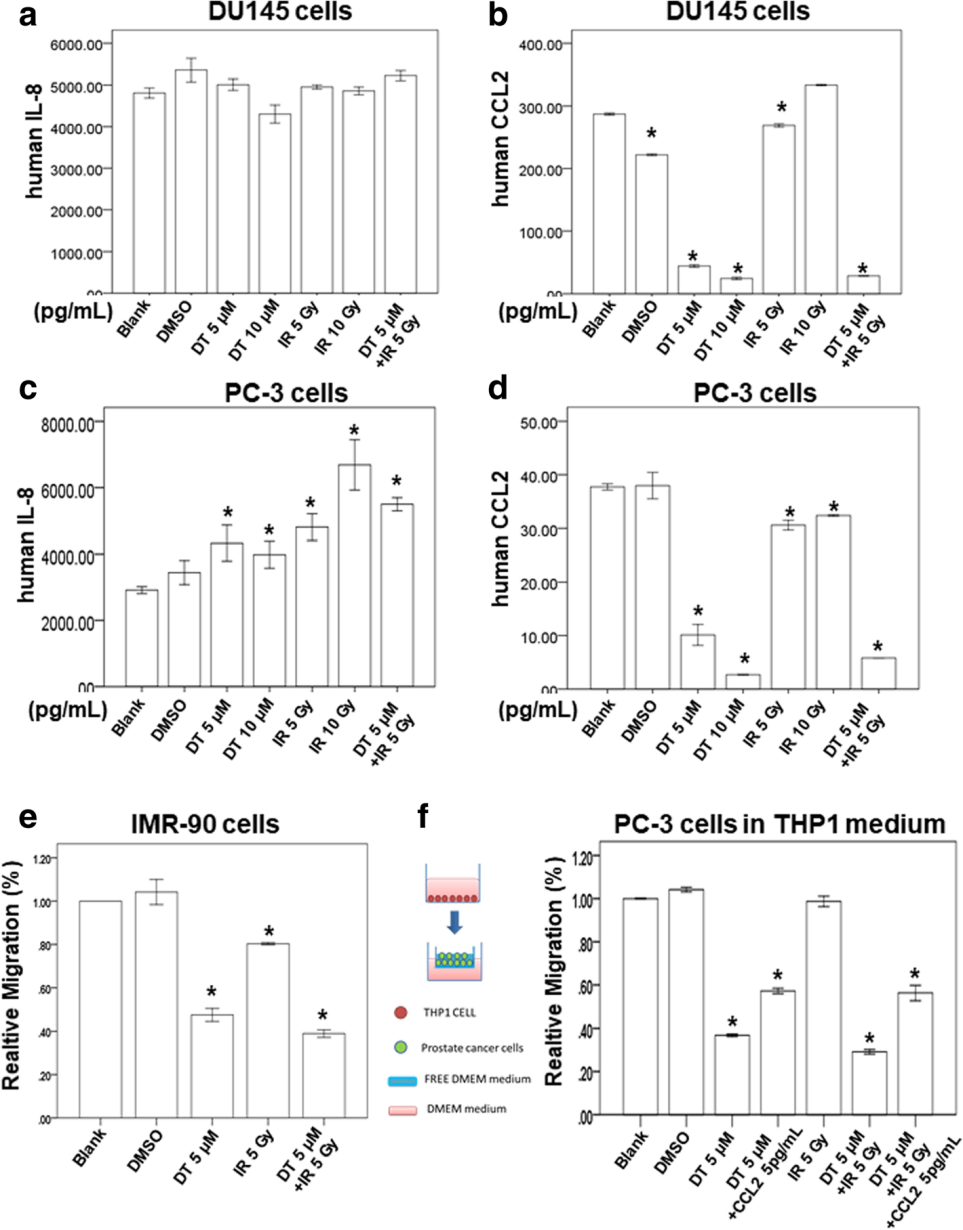
Effects of DT on the protein secretion of IL8 or CCL2 from prostate cancer cells and on human normal fibroblasts migration in the THP1 cell medium. The condition medium of coculture with DU145 cell or PC-3 cells were collected from untreated cells, cells treated with DMSO or indicated treatment for 24 h. The secretion of human IL8 was measured with ELISA kits (**a**, **c**). The secretion of human CCL2 was measured with ELISA kits (**b**, **d**). **e** The migration ability of IMR-90 cells were measured with the transwell migration assay. After treated with indicated drugs for 24 h, the photographs (× 100) were taken and the migratory cells were measured using AlphaEase®FC StandAlone Software. Numbers of the migratory IMR-90 cells in each group were normalized to the control. **f** The migration ability of human prostate cancers in the macrophages medium were measured by the transwell migration assay. THP1 cells were treated with DMSO or DT for 24 h. Then the conditioned medium was collected and placed in the lower chamber. In the group of DT+ 5 pg/mL CCL2, 5 pg/mL CCL2 was added into the condition medium of this group. Then, PC-3 cells were then placed on the upper chamber for the migration assay. After incubation for 24 h, the photographs (× 100) were taken and the migratory cells were measured using AlphaEase®FC StandAlone Software. The quantification of the indicated migratory cells numbers in each group were normalized to the control. All the results are representative of at least three independent experiments. (Error bars = mean ± S.E.M. Asterisks (*) mark samples significantly different from blank group with *p* < 0.05)

Our results indicated that DT inhibits the motility of prostate cancer cells, even in the THP-1 cell medium. In previous studies, increased CCL2 expression in prostate cancer cells encouraged metastasis through macrophage recruitment [18–20]. In another study, IL-8 was demonstrated to be an important molecule for androgen-independent prostate cancer growth and progression [21]. We subsequently used ELISA to examine the effects of different treatments on the secretion of IL-8 or CCL2 by prostate cancer cells. After conducting the indicated treatment for 24 h, we found that IL-8 secretion was not inhibited (Fig. 3a, c). However, DT and combined treatment with IR (5 μM DT plus 5 Gy IR) considerably inhibited the secretion of CCL2 by prostate cancer cells (Fig. 3b, d). To validate the critical role of CCL2 in controlling the migration ability of DT-treated prostate cancer cells, we investigated the effects of DT or combined treatment with IR (5 μM DT plus 5 Gy IR) and with or without CCL2 on the migration ability of prostate cancer cells. After adding 5 pg/mL of CCL2 to the conditioned medium, we observed that CCL2 partially rescued the migration ability of DT- or combination-treated PC-3 cells (relative migration: from 30 to 40% to 60%) (Fig. 3f). Our results suggest that CCL2 is among the cytokines that control the migration ability of DT- or combination-treated prostate cancer cells, and that DT and combined treatment inhibit the migration of prostate cancer cells through their effects on CCL2. In our previous study, we found that the proliferation of IMR-90 cells (normal human lung fibroblasts) was mildly inhibited by 5 μM DT treatment for 24 h [22]. In addition, we discovered that 5 μM DT inhibits the migration ability of IMR-90 cells (Fig. 3e). The migration ability of IMR-90 cells was mildly inhibited by 5 Gy IR. In the present study, combination treatment with 5 μM DT and 5 Gy IR was also able to inhibit the migration ability of prostate cancer cells. These data suggest that DT and the combined therapy can inhibit the migration ability of both prostate cancer cells and normal human lung fibroblasts.

### Effects of DT on cell cycle progression of prostate cancer cells in vitro

**Fig. 4:**
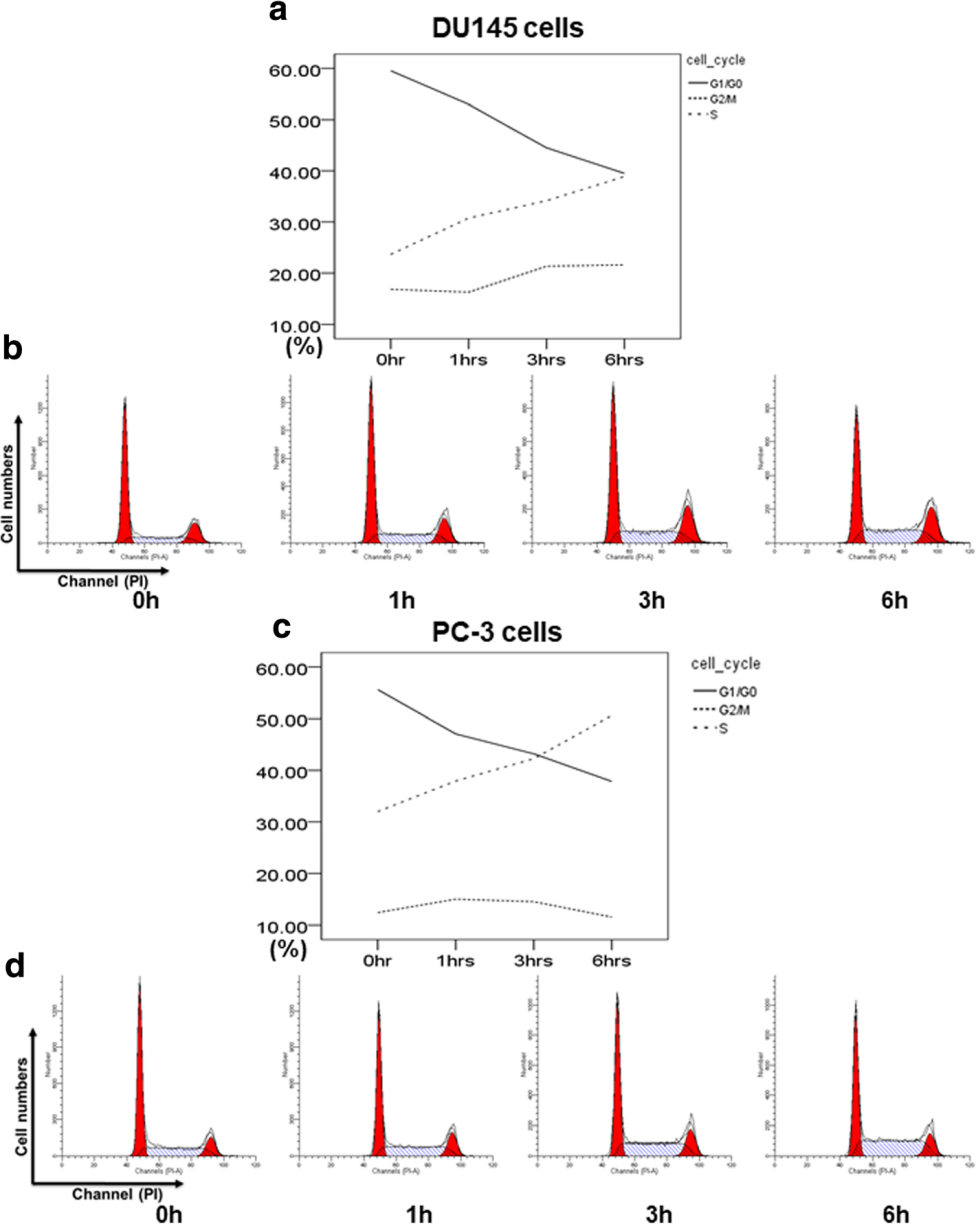
Effects of DT on human prostate cancer cell cycle. DU145 cells or PC-3 cells were treated with 10 μM of DT for 0, 1, 3, 6 h and then subjected to flow cytometry with PI staining. The cell cycle phase distribution of DU145 cells (**a**) or PC-3 cells (**c**) after treatment with DT were measured in the indicated hours. The representative histograms between PI intensity and cell numbers of DU145 cells (**b**) or PC-3 cells (**d**) were measured by flow cytometry

Because DT demonstrated potential antimigration effects on prostate cancer cells, we further investigated the mechanism of DT on prostate cancer cells. First, we determined the effect of DT on the cell cycle progression of prostate cancer cells. Compared with the control cells, the S-phase proportion of cells treated with 10 μM DT increased from 30% to 40–50% in 6 h (Fig. 4). The results showed that DT treatments were able to prolong or arrest the S phase of the prostate cancer cell cycle.

### DT regulates DDR proteins in prostate cancer cells

**Fig. 5:**
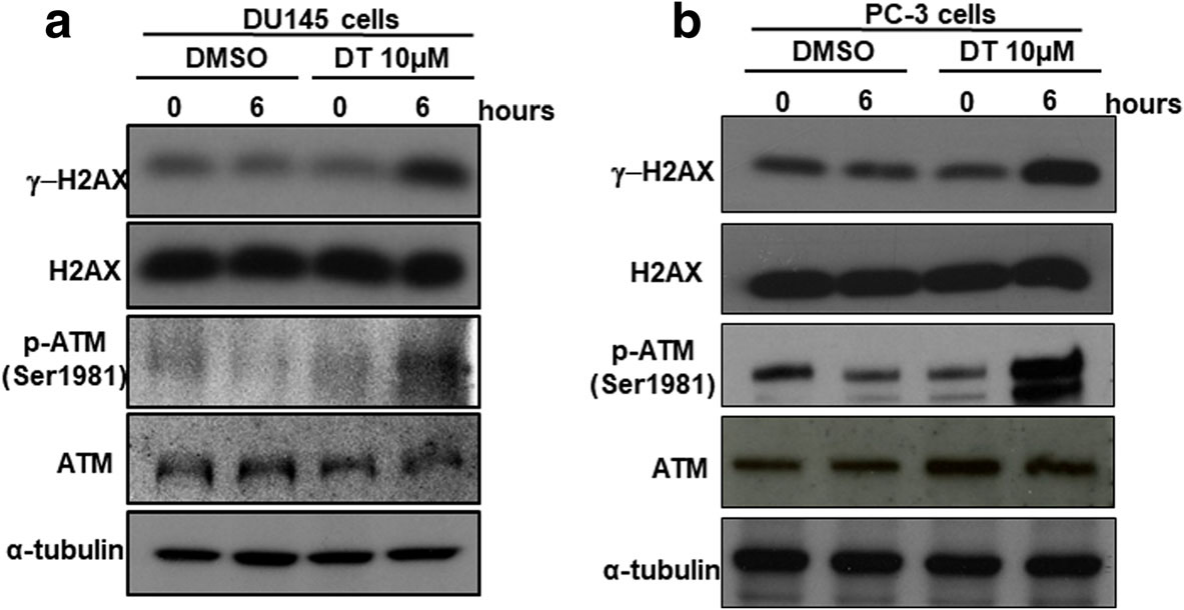
DT inhibits the protein expression of DNA damage response proteins in prostate cancer cells. Total cell extracts of DU145 cells (**a**) or PC-3 cell (**b**) were harvested from untreated cells and cells treated with DMSO or DT for 0, 6 h. The protein was immunoblotted with polyclonal antibodies specific for DNA damage response proteins (γ-H2AX and phosphorylated-ATM). α-tubulin was used as an internal loading control

During DDBs, ATM is autophosphorylated at serine 1981 and phosphorylates H2AX at serine 139, which then recruits downstream repair proteins to DNA damage foci for repair. In this stage, the S phase can be delayed. Several cellular factors that are directly phosphorylated by ATM contribute to the S-phase checkpoint. Considering how the previous data showed that DT may prolong the S phase of the cell cycle in prostate cancer cells after 6 h, we used a western blot assay to investigate its effect on these DNA damage proteins, phosphorylated (S1981)-ATM (p-ATM) and 푟H2AX, in DU145 cells and PC-3 cells. We found that 10 μM DT induced the protein expression of 푟H2AX and p-ATM in DU145 cells (Fig. 5a) or PC-3 cells (Fig. 5b) within 6 h. These data suggested DT has the potential to induce DDR in prostate cancer cells.

## Discussion

In previous studies, various members of the tanshinone family were demonstrated to be capable of inhibiting the growth of several types of cancer cells, including prostate cancer cells [23–25]. Moreover, tanshinone IIA can suppress the migration ability of osteosarcoma MG-63 cells and gastric cancer AGS cells [26, 27]. Another recent study showed that 15,16-dihydrotanshinone I has a structure similar to that of DT and induces endoplasmic reticulum stress in prostate carcinoma cells (DU145 cells) [28]. Tanshinone IIA and DT possess an ortho-quinone and an intact ring D, respectively, suggesting that the structure of tanshinones may influence the ways in which they inhibit migration ability in tumors. In our previous study, we also found that DT substantially inhibited the migratory ability of prostate cancer cells in both a macrophage-conditioned medium and macrophage/prostate cancer coculture medium [12]. In this study, we discovered that the combination treatment with 5 μM DT and 5 Gy IR more strongly inhibited the migration ability of prostate cancer cells compared with 5 μM DT (Figs. 1 and 2). These results suggest that DT cooperates with radiation therapy and demonstrate the effectiveness of the combined therapy in inhibiting the migration of prostate cancer cells.

CCL2 regulates macrophage differentiation, growth, and chemotaxis [29]. Furthermore, CCL2 was previously shown to inhibit the generation of tumor-reactive T cells [30]. Tanshinone IIA was found to have considerable growth-inhibiting effects on U-937 cells through the induction of apoptosis by downregulation of CCL2 expression [25]. In our previous studies, DT reduced the secretion of CCL2 by both lung cancer cells and prostate cancer cells [12, 22], yet the effect of the combined therapy on CCL2 secretion by prostate cancer cells is unclear. In the present study, we discovered that IR 5 Gy mildly decreased CCL2 secretion by both PC-3 and DU145 cells. Moreover, the combination treatment inhibited CCL2 secretion by prostate cancer cells more strongly than 5 μM DT did (Fig. 3 b and d). These results suggest that DT cooperates with radiation therapy to affect CCL2 secretion. The previous report showed that tanshinone IIA inhibited cyclic strain-induced IL-8 expression by the induction of heme oxygenase (HO-1) in endothelial cells [31]. Tanshinone I also reduced the transcriptional activity of IL-8 [32]. In our results, DT did not decrease the secretion of IL-8 by either PC-3 or DU145 cells. In a previous study, fluorouracil, which interrupts the action of this enzyme and blocks synthesis of the pyrimidine thymidine, increased the levels of IL-8 secreted from PC-3 cells [33]. Notably, increased IL-8 expression was also observed after treatment with DT only, IR only, or combined therapy in PC-3 cells (Fig. 3c). These results suggest that the mechanism of DT on PC-3 cells may occur at the DNA level. By contrast, a previous study showed that the structural difference between several tanshinones at the C-15 position of the furan ring resulted in differential inhibition of CYP3A (cytochrome P450 3A) activity [34]. The positioning of the furan ring in DT and tanshinone IIA might have caused this difference. The underlying mechanism will be investigated in our future research.

Among the various types of DNA damage, the most deleterious is DNA DDBs. Double-strand breaks can be generated using exogenous sources, including IR or chemotherapeutic agents. During DDBs, ATM is phosphorylated and then phosphorylates histone H2AX at S139 (known as γH2AX). If they remain unrepaired, DDBs may induce cell death and apoptosis [35]. Numerous chemotherapeutic agents and types of radiotherapy exert cytotoxic effects by inducing DNA DDBs. According to previous studies, tanshinone IIA can bind to the DNA minor groove [36] and significantly sensitize oral squamous cell carcinoma to radiation through the autophagy pathway [37]. In one report, 5–20 μM tanshinone IIA combined with IR 6Gy was able to induce γH2AX expression. However, the effect of tanshinone monotherapy on the DNA damage pathway is still unclear. This is the first study to demonstrate that 10 μM DT increased the S phase proportion of the cell cycle and phosphorylated ATM and γH2AX expression in prostate cancer cells. The results suggest that DT can induce DNA DSBs and increase the effect of IR.

In one meta-analysis of patients with locally advanced prostate cancer, optimal oncological outcomes were obtained through radical prostatectomy with adjuvant radiotherapy or radiotherapy with adjuvant hormonal therapy [38]. These results suggest that radiotherapy is a vital treatment for locally advanced prostate cancer. However, radiotherapy can increase the risk of urinary incontinence, bladder neck contracture, and urethral stricture [39, 40], and can cause capillary destruction with reduced vascularity and tissue fibrosis [39]. To improve outcomes, in several countries radiotherapy is often combined with complementary and alternative medicine [41–43]. Notably, tanshinone IIA not only sensitizes oral squamous cell carcinoma to radiation through the autophagy pathway [37], but also has a protective effect against radiation-induced ototoxicity in HEI-OC1 cells, a mouse auditory cell line [44]. Sodium tanshinone IIA sulfonate has also been shown to prevent radiation-induced toxicity in cardiomyocytes [45]. These reports suggest that tanshinones have different effects on radiation therapy in different cell lines. However, the effects and mechanisms of these combined therapies on the immune system are still unclear. Our results first demonstrated that combined treatment (5 μM DT with 5 Gy IR) was able to diminish migration of prostate cancer cells in a more effective manner than did the larger dose of IR (10 Gy). The combination treatment reduced CCL2 in prostate cancer cells. In addition, the combined treatment decreased the ability of macrophages to recruit prostate cancer cells. In our previous study, we also discovered that DT inhibited the secretion of CCL2 by the cultured medium of macrophages, including THP-1 cells and RAW 264.7 cells [12]. These results suggest that macrophages and cytokines, like CCL2, may regulate the effect of combined treatment on prostate cancer.

## Conclusions

In conclusion, DT displays radiosensitization and antimigration effects in prostate cancer cells by inducing DNA damage and inhibiting CCL2 secretion. We suggest that DT can be used as a novel antimetastatic cancer drug or radiosensitizer in the armamentarium of prostate cancer management.

## Abbreviations

ATM: Ataxia telangiectasia-mutated
CCL2: Chemokine C-C Motif Ligand 2
DDBs: DNA double-strand breaks
DDR: DNA damage response
DMEM: Dulbecco’s Modified Eagle’s medium
DMSO: Dimethyl sulfoxide
DT: Dihydroisotanshinone I
ELISA: Enzyme-linked immunosorbent assay
FBS: Fetal bovine serum
MDC1: Mediator of DNA damage checking protein 1
PVDF: Polyvinylidene difluoride
SDS-PAGE: Sodium dodecyl sulfate polyacrylamide gel electrophoresis
SEM: Standard error of mean
TBST: Tris-Buffered Saline Tween-20
